# Understanding the evolutionary relationships and major traits of *Bacillus *through comparative genomics

**DOI:** 10.1186/1471-2164-11-332

**Published:** 2010-05-26

**Authors:** Luis David Alcaraz, Gabriel Moreno-Hagelsieb, Luis E Eguiarte, Valeria Souza, Luis Herrera-Estrella, Gabriela Olmedo

**Affiliations:** 1 Departamento de Ingeniería Genética, Centro de Investigación y de Estudios Avanzados del I.P.N. Campus Guanajuato, AP 629 Irapuato, Guanajuato 36500, México; 2 Department of Biology, Wilfrid Laurier University, 75 University Ave. W. Waterloo, ON, N2L 3C5, Canada; 3 Departamento de Ecología Evolutiva, Instituto de Ecología, Universidad Nacional Autónoma de México, CU, AP 70-275 Coyoacán 04510 México DF; 4 Laboratorio Nacional de Genómica para la Biodiversidad (Langebio), Centro de Investigación y de Estudios Avanzados del I.P.N. Campus Guanajuato, AP 629 Irapuato, Guanajuato 36500, México

## Abstract

**Background:**

The presence of *Bacillus *in very diverse environments reflects the versatile metabolic capabilities of a widely distributed genus. Traditional phylogenetic analysis based on limited gene sampling is not adequate for resolving the genus evolutionary relationships. By distinguishing between core and pan-genome, we determined the evolutionary and functional relationships of known *Bacillus*.

**Results:**

Our analysis is based upon twenty complete and draft *Bacillus *genomes, including a newly sequenced *Bacillus *isolate from an aquatic environment that we report for the first time here. Using a core genome, we were able to determine the phylogeny of known *Bacilli*, including aquatic strains whose position in the phylogenetic tree could not be unambiguously determined in the past. Using the pan-genome from the sequenced *Bacillus*, we identified functional differences, such as carbohydrate utilization and genes involved in signal transduction, which distinguished the taxonomic groups. We also assessed the genetic architecture of the defining traits of *Bacillus*, such as sporulation and competence, and showed that less than one third of the *B. subtilis *genes are conserved across other *Bacilli*. Most variation was shown to occur in genes that are needed to respond to environmental cues, suggesting that *Bacilli *have genetically specialized to allow for the occupation of diverse habitats and niches.

**Conclusions:**

The aquatic *Bacilli *are defined here for the first time as a group through the phylogenetic analysis of 814 genes that comprise the core genome. Our data distinguished between genomic components, especially core vs. pan-genome to provide insight into phylogeny and function that would otherwise be difficult to achieve. A phylogeny may mask the diversity of functions, which we tried to uncover in our approach. The diversity of sporulation and competence genes across the *Bacilli *was unexpected based on previous studies of the *B. subtilis *model alone. The challenge of uncovering the novelties and variations among genes of the non-*subtilis *groups still remains. This task will be best accomplished by directing efforts toward understanding phylogenetic groups with similar ecological niches.

## Background

*Bacillus *is one of the best characterized bacterial genera. Since the late 19^th ^century, the long history of Bacilli research has included classical microbiology, biochemistry, and more modern genomic and proteomic approaches. *Bacillus *is defined as a Gram-positive, rod-shaped bacterium that can be aerobic or facultative anaerobic [1] and produces highly resistant dormant endospores in response to nutritional or environmental stresses [2].

*Bacilli *are ubiquitous bacteria that exploit a wide variety of organic and inorganic substrates as nutrient sources [1]. However, spore dispersal by air and water [3] may lead to false conclusions about the ecological significance of recovered bacillus isolates, since it is not clear if the robust presence of the bacteria is due to the resistant nature of the dispersed spores or due rather to a large adaptive capacity that would allow the bacteria to be found in an active, vegetative state in diverse environments [2]. This study of the *Bacillus *pan-genome, and in particular the functional categorization of the accessory genomes and their relationship or lack thereof with the environment, may provide insight into these important biological questions.

There are several ways to classify this group according to biochemistry, lifestyles, and/or growth on different substrates. One classification of the *Bacillus *splits them into three major classes [1]: pathogenic, environmental, and those used for industrial purposes. The pathogenic class is represented by *B. anthracis, B. cereus, and B. thuringiensis*. Environmental Bacilli are quite diverse and include *B. subtilis, B. pumilus, B. halodurans*, and *B. coahuilensis*. The strain *B. licheniformis *is a well known representative of an industrial strain [1]. This classification is useful for introducing the metabolic diversity of the genus, but it provides no guidance on a phylogenetic classification of the *Bacillus *for research purposes. In addition, this type of classification does not consider any aquatic *Bacillus*. There is need for a classification method that could take advantage of the nearly 130 genome projects of the genus. With more than 108 complete and draft genome sequences available to date, *Bacillus *is one of the most represented genera in the genomic databases.

Although there are close to 1,000 complete prokaryotic genome sequences to date, the group is highly biased toward pathogenic isolates [4,5]. Among 85 *Bacillus *genomes, 61% are devoted to the cereus-*anthracis*-*thuringiensis *group (See Additional file 2: Table S1). Several researchers have used this overrepresentation as an advantage to perform comparative genomic studies aimed at defining the population structure and finding genetic markers for pathovar identification [6-9]. Despite the oversampling of pathogens, genomes of *Bacillus *isolated from a wide range of environments are available, including hydrothermal vents [10], tidal flats [11], soil [12], alkaline environments [13,14], shallow marine water [15], and a shallow water column from an oligotrophic environment [16]. The presence of *Bacillus *in these different environments reflects the broad metabolic capabilities of a widely distributed genus.

In a report of the intra-species diversity of *Streptococcus agalactiae *[17], the "pan-genome" concept was defined as the sum of the core genome (comprising genes present in all analyzed strains) and the "accessory" genome (comprising all strain-specific genes) [18,19]. This concept has been expanded for comparisons at other taxonomic levels, such as family [20,21] and for defining the universal ancestor hypothetical core [22]. Most traditional markers for species identification, such as 16S rRNA genes, Comparative Genome Hybridization (CGH), and the classical measures of phenotypic similarity, mask the real genetic diversity since they rely mainly on core-genome genes [17,23,24]. Another approach to unveil the microbial diversity used mostly by population geneticists and by clinical microbiologists are the Multi Locus Sequence Typing (MLST) and Multi Locus Sequence Analysis (MLSA) methods [25] relying on the analysis of internal fragments of housekeeping genes (usually 7 genes), which are useful for understanding populations dynamics, recombination, and pathogen diagnosis. Phylogenetic relationships can be obtained through more extensive genomic sampling, such as the one afforded by the genome sequences. Analyzing the whole set of conserved genes across a taxonomical level, such as the core genomes, will shed light about evolutionary and functional relationships among the related species. Several methods based on pairwise ortholog comparison and synteny strategies have been developed to assess the composition of core genomes [17,20,23].

In this study, we were interested in understanding the cohesion of the *Bacillus *genus at the genomic level by using the core and pan-genomes as the working units and taking advantage of the large dataset available. We have recently described the complete genome of *Bacillus coahuilensis *[16,26], which possesses one of the smallest genomes (3.35 Mb; 38% GC) reported for a free-living bacteria in the group, and have identified genes that allow this bacterium to survive in an aquatic oligotrophic environment. We now report the genome sequence of another isolate, *Bacillus *sp. m3-13 with a genome size of 4.13 Mb from the same environment as *B. coahuilensis*. We compared these genomes to ask whether the common environment has selected for similar features in the two genomes. These coincidences would be observed in their gene constitutions as cohesion of similar classes of metabolic genes.

**Figure 1:**
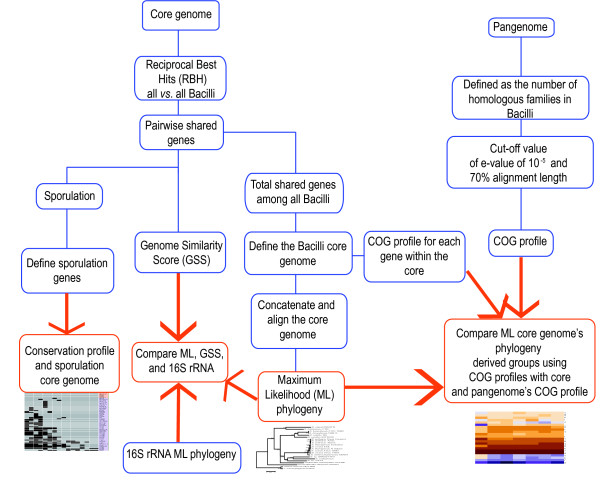
**Overview of this work**.

To obtain insight into the group's biology we describe the relatedness within the *Bacillus *using whole genome information to reconstruct their evolutionary history taking advantage of the dataset available from the complete and draft genomes of 20 Bacillus isolated from a wide range of environments. We compared the use of different conserved genes as well as pairwise shared genes to address local phylogenies and measure quantitatively the relatedness between species using the core genome. Analysis of the functional categories of the core and pan-genome revealed a clear separation between different groups and reflected the niche of the *Bacillus *strains. Finally, clustering of conserved/absent genes for competence and sporulation genes, distinctive processes of the *Bacillus *genus, showed that genetic mechanisms for sporulation are far more diverse across *Bacillus *than expected from studies of *B. subtilis *alone (Figure 1).

## Results

### *Bacillus *sp. m3-13 genome summary

*Bacillus *sp. m3-13 genome was sequenced using a 454 FLX system (454 Life Sciences) with a 20-fold coverage. Assembly of the sequences resulted in 50 contigs and a total of 4,137,575 bp assembled. The entire genome is 4.1 Mb with 40% GC content, 4,417 coding sequences, and 81 RNAs.

*Bacillus *sp. m3-13 was isolated from a desiccation lagoon in Cuatro Cienegas, Coahuila, Mexico within the same sampling of the previously described *B. coahuilensis *strain [16,26]. Using a 16S rRNA comparison, we determined that the *B. horikoshii *NBSL26 is the closest strain to *Bacillus *sp. m3-13 with 99% identity. Both *B. coahuilensis *and *Bacillus *sp. m3-13 live in the same oligotrophic environment, which includes low phosphorous levels (less than 0.3 μM), but they appear to have different strategies for dealing with the poor nutrient environment. *B. coahuilensis *produces sulfolipids and replaces membrane phospholipids [16], In contrast, *Bacillus *sp. m3-13 has *phn *genes, which code for phosphonate ABC importers, permeases, and a phosphonate-lyase, and we hypothesize that the strain may use these genes to take up and assimilate phosphonates. Both strategies seem to be used by bacteria from the Cuatro Cienegas [27], as well as by marine bacteria [28,29].

### *Bacillus *core genome phylogenetics

To gain insight into the natural history of *Bacillus *based on the complete genome sequence, we performed a phylogenetic reconstruction using all of the core genes. We chose at least one representative from each major division within the Bacillaceae family to maintain the metabolic cohesion behind the diverse ecological groups. The type of strains used for this analysis as well as their general genome features, habitats, accession numbers, isolation data, and outstanding phenotypes are summarized in Table 1.

**Table 1:** Species, accession numbers, and general features of *Bacillus *sp. used in this work [30]

Strains	Accession	CDS	GC%	Habitat	Phenotype	Isolation environment	Reference
*Bacillus *sp. m3-13	ACPC00000000 (WGS semi-finished)	4294	40	Fresh water	N/A	Chihuahuan desert lagoon in Cuatro Cienegas, Coahuila, Mexico in 2005	This work
*Bacillusc oahuilensis *m4-4	NZ_ABFU00000000 (WGS semi-finished)	3642	38	Fresh water	N/A	Chihuahuan desert lagoon in Cuatro Cienegas, Coahuila, Mexico in 2005	[16]
*Bacillus subtilis *subsp. *subtilis *str. 168	NC_000964	4408	43.5	Soil	N/A	X-ray irradiated strain in Marburg in 1947	[12]
*Bacillus halodurans *C-125	NC_002570	4326	43.7	Soil, Fresh water	Alkalophile	1977	[14]
*Bacillus cereus *ATCC 10987	NC_003909	6248	38	Dairy isolate, Soil	Pathogen	Cheese spoilage in Canada	[31]
*Bacillus anthracis *str. Ames	NC_003997	5569	35.4	Soil	Non-Pathogen	N/A	[32]
*Oceanobacillus *iheyensis HTE831	NC_004193	3736	35.7	Marine	Alkalophile	Deep sea mud at 1050 m depth from the Iheya ridge near Okinawa Japan in 1998	[11]
*Bacillus cereus *ATCC 14579	NC_004722	5610	35.3	Soil	Pathogen	N/A	[9]
*Bacillus anthracis *str. Sterne	NC_005945	5641	35.4	Soil	Non-Pathogen	N/A	Unpublished
*Bacillus thuringiensis *serovar konkukian str. 97-27	NC_005957	5590	35.4	Host, Soil	Pathogen	Severe human tissue necrosis	[33]
*Bacillus licheniformis *ATCC 14580	NC_006270	4371	46.2	Soil	Pathogen, Subtilisin production, Amylase production	N/A	[13]
*Bacillus cereus *E33L	NC_006274	6010	35.4	Soil	Pathogen	Swab of a zebra carcass in Ethosha National Park in Namibia in 1996	[33]
*Geobacillus kaustophilus *HTA426	NC_006510	3733	52.1	Deep sea, Marine	N/A	N/A	[10]
*Bacillus clausii *KSM-K16	NC_006582	4349	44.8	Soil	Alkalitolerant, Probiotic, Protease production	N/A	Unpublished
*Bacillus anthracis *str. 'Ames Ancestor'	NC_007530	5973	35.4	Soil	Pathogen	N/A	[34]
*Bacillus thuringiensis *str. Al Hakam	NC_008600	5090	35.4	Host, Soil	Pathogen	Severe human tissue necrosis	[35]
*Bacillus pumilus *SAFR-032	NC_009848	3913	41.3	Soil	Biomass degrader, Pathogen, Radiation resistant	Spacecraft Assembly Facility at NASA Jet Propulsion Laboratory	[36]
*Bacillus weihenstephanensis *KBAB4	NC_010184	6133	35.4	Soil	Non-Pathogen	N/A	Unpublished
*Bacillus *sp. NRRLB14911	NZ_AAOX00000000 (WGS semi-finished)	5869	45.7	Marine	N/A	10 meters depth in the Gulf of Mexico	[15]

As noted in the literature, the total number of genes within a core-genome tends to diminish as more genomes from related strains are incorporated into the analysis [5,17,23]. *B. coahuilensis *has the smallest genome reported for a *Bacillus *and thus is a good reference to address the number of genes shared between all of the representatives of *Bacillus*. A full matrix of Reciprocal Blast Hits (RBH) was constructed for the identification of the 814 orthologous genes shared by all 20 species analyzed and was defined as the core genome. The average gene content for *Bacillus *was 4,973 ± 923 genes and thus the core genome reflected only a fifth of the total content of an average genome. In contrast, after clustering homologous protein families, an estimated pan-genome size of 155,747 genes was obtained. All pan-genome genes were grouped into 19,043 families and reflect the large repertoire of genes within this cosmopolitan group.

**Figure 2:**
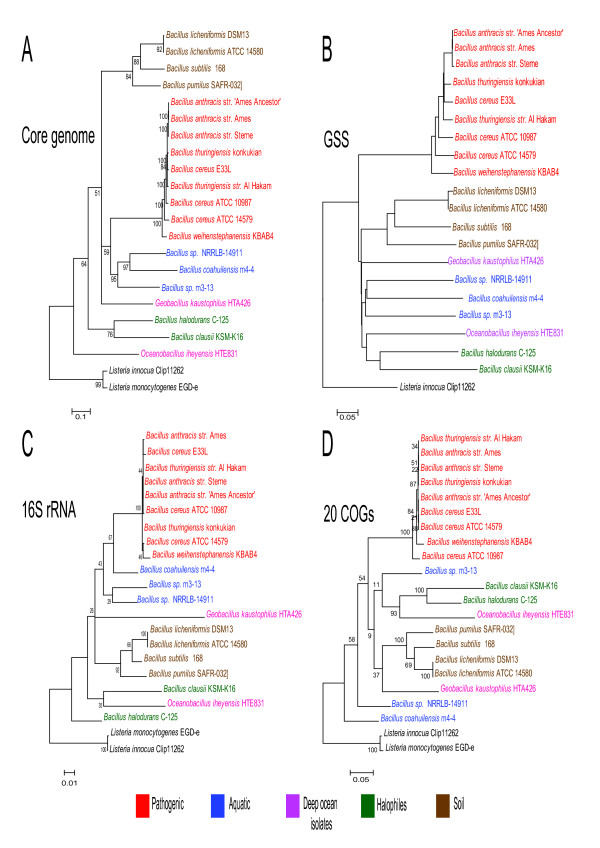
**Phylogenetic reconstruction for *Bacillus***. **A**. Concatenated 814 translated genes of the core genome maximum likelihood (ML) phylogeny. Bootstrap values are shown. **B**. Genome Similarity Score (GSS) distance matrix plotted as a Neighbor-Joining tree. **C**. ML phylogeny using 16S rRNA. **C**. Concatenated 20 Conserved Universal Cogs (uCOGs) ML phylogeny. Note how inner groups are well defined and supported only in the core genome phylogeny (**A**) and how the GSS distance (**B**) resembles the inner groups described in the core's genome phylogeny.

We reconstructed a Maximum Likelihood (ML) phylogeny using concatenated alignments of the 814 translated core genes across the 20 species (Additional file 1), resulting in a 308,782 amino-acid length alignment (Figure 2A). The phylogeny created clusters of the following major groups: *B. clausii-halodurans, B. subtilis-licheniformis-pumilus, B. anthracis-thuringiensis-cereus*, and a novel group, *Bacillus *sp. NRRLB-14911-*coahuilensis*-m3-13, The strain *Geobacillus kaustophilus*is within the major groups in its own leaf but deep into the *Bacillus*, whereas *Oceanobacillus iheyensis *is basally located outside the major groups. A sister group is formed by *B. halodurans-clausii *falling on the edge of the main *Bacillus *groups. Another distinctive feature of the core genome phylogeny, as compared with the traditional 16S rRNA and the universally conserved COG phylogenies, is its robustness that is reflected by the generally high bootstrap replica values. Still, this tree is not fully resolved, as shown by the position of the *G. kauustophilus *leaf.

We further measured the distance between species with a Genomic Similarity Score (GSS) [37]. This measurement is based on the sum of bit-scores of shared orthologs, detected as RBH, and normalized against the sum of bit-scores of the compared genes against themselves (self-bit-scores). It has a range from 0 to 1 with a maximum reached when two compared proteomes are identical. There is an average of 2,539 ± 561 shared genes between different *Bacillus *species and an average GSS of 0.5637 ± 0.0039. A distance matrix of the GSS scores of the shared orthologs for the 20 Bacillus groups was plotted in a Neighbor-Joining tree to evaluate the resolution of a pairwise, shared orthologs index as an evolutionary distance tool (Figure 2B). All of the inner groups shown in the core genome phylogeny appear in this clustering, although inner sister groups are clustered showing the ambiguity of the deep nodes. GSS can therefore be used as a complementary approach, as an index to clarify relationships among organisms using whole pairwise shared orthologs. GSS takes into account the maximum comparable pairwise genome shared, in contrast to the regular phylogenetic reconstruction, which can only compare a common dataset of homologous genes.

A 16S rRNA Maximum Likelihood phylogeny of the selected strains is shown in Figure 2C. Major groups, such as the *B. cereus *and *B. subtilis*' groups, are maintained with low bootstrap support (< 50), while the aquatic *Bacillus *group is paraphyletic within this phylogeny. The use of 20 conserved concatenated Cluster of Orthologous Groups (COGs) as described in [38-40] for *Bacillus *(5,299 amino acid alignment length) to perform an ML phylogeny (Figure 2D) resulted in a tree that shows also a paraphyletic aquatic *Bacillus *group and lower bootstrap support when comparing inner groups like *B. cereus *group to the core genome phylogeny. We noted that the 16S phylogeny places *G. kaustophilus *as internal clade within the *Bacillus *genus, but with considerable substitution rates shown in the large branch length, while *B. clausii *and *O. iheyensis *are placed in the same clade though with very low bootstrap support; in contrast, the conserved universal COGs (uCOGs) phylogeny places *O. iheyensis *close to a branch formed by *B. halodurans *and *B. clausii*. Neither the 16S rRNA nor the uCOGs resolve the internal clades with as much support as the core genome phylogeny.

### Functional composition of the core genome of sequenced *Bacillus*

**Figure 3:**
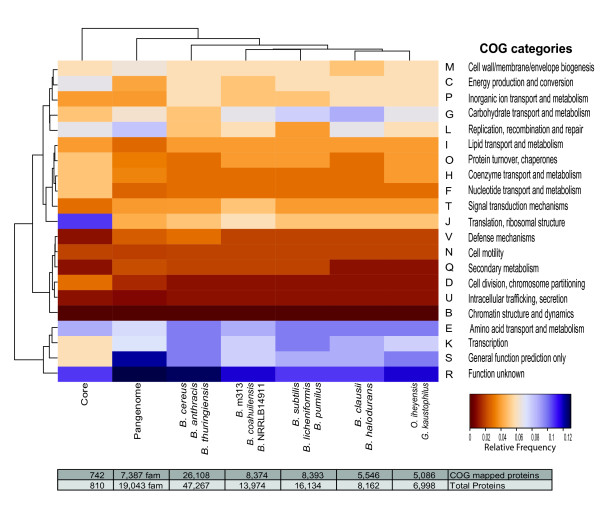
**Heat map comparison of the core genome, pan-genome, and *Bacillus *groups conservancy of COGs**. Normalized Cluster of Orthologous Groups (COGs) frequencies within each group are shown as a two way clustering; interestingly the clustering supports the groups formed in the core genome phylogeny (horizontal axis). In vertical axis COGs are grouped according to their abundance showing moderate conserved COGs in the top, low frequency conserved COGs in the central clusters and highly abundant COGs cluster in the lower clusters. In the bottom total numbers of proteins families mapped to COG within each row are shown.

To understand the functional roles of the genes that constitute the core and pan-genome, we took advantage of the COGs functional classification [41]. This is a classification system based on orthologous relations among genes. We used the COGs to map the core genome, pan-genome, and each of the four groups defined in the core phylogeny (Figure 3): i) *B. anthracis-cereus-thuringiensis*, ii) *B. subtilis-licheniformis-pumilus*, iii) *B. clausii-halodurans*, iv) *B. coahuilensis*-m313-nrrlb14911, v) *G. kaustophilus-O. iheyensis*. These last two do not form a phylogenetically related group, however, for the functional analysis we chose to group them given the similarities in the environments from which these strains were recovered. Using this strategy, we grouped *Bacillus *representatives that had shared evolutionary and ecological features and then searched for over/under represented gene functions within each group to underscore relevant gene functions for each evolutionary group. We observed significant differences in the COGs categories of core-pan-genome-groups (Chi square = 753.72; d.f. = 126; p-value < 2.2e^-16^). Figure 3 shows a heat plot map of the ratio of normalized genes to total gene content for each COG category. Several expected features arose from this analysis, such as the predominance of genes in COG category J (translation and ribosomal structure genes; core = 0.11/average = 0.06) within the core. This was expected as most of the conserved universal COGs are contained within this category [40]. Their conservation across taxa and functional restrains are precisely why they are chosen as gold standards for phylogenetics in addition to 16S rRNA genes. Other over-represented categories, although only within the core, are C (energy production and conversion; core = 0.07/average = 0.06) and L (replication, recombination, and repair; core = 0.07/average = 0.05). Highly represented categories included COGs E (amino acid transport and metabolism; core = 0.09/average = 0.1), R (function unknown; core = 0.11/average = 0.12), and S (poorly characterized genes; core = 0.06/average = 0.09) that were not only in the core but also in the pan-genome and in all of the *Bacillus *groups. Interestingly, several core genes of the COG R and S are conserved across the entire *Bacillus *with some clearly being involved in the sporulation process, including *spmA, spmB, yaaT, spoIVFB, spoVB, spoVR*, and others (See Additional file 2: Table S2).

Some categories are at least partially over-represented in the core when compared to the pan-genome and to either group and include the following: COG H (coenzyme transport and metabolism; core = 0.05/pan-genome = 0.03), which includes biosynthesis genes for biotin (*birA*), riboflavin (*ribA*), co-enzyme A (*ylol*), and a dipicolinate synthase (*spoVFB*); COG F (nucleotide transport and metabolism; core = 0.05/pan-genome = 0.03), which includes *comEB *competence protein; COG O (protein turnover and chaperones; core = 0.05/pan-genome = 0.03), which includes genes such as *groES*; and COG D (cell division), which includes the DNA translocase *ftsK*, the rod shape-determining gene *rodA*, and some sporulation-related genes such as *spoIID*, *spoVE*, and *soj*.

COG categories U (intracellular trafficking and secretion), N (cell motility), and I (lipid transport) are in the same range and the fractional differences between the core, pan-genome, and *Bacillus *groups of these categories are not noticeable (average = 0.01). Categories K (transcription; core = 0.06/average = 0.09) and S (general functions; core = 0.06/average = 0.09) are underrepresented in the core. Despite the ubiquity of these genes within *Bacillus*, there are only a few transcription-related proteins that are shared between all of them. It has been shown, however, that a given transcription factor ortholog selected solely by RBH may not have a conserved function, and this aspect depends on the phylogenetic distance and tempo of the rapidly evolving regulatory networks [42]. We expected and observed important differences in this category, since the fine tuning of gene expression reflects the far and wide distribution of metabolic diversity in Bacillus. We also expected an underrepresentation of genes in COG T (signal transduction; core = 0.03/average = 0.04) and COG V (defense mechanisms; core = 0.01/average = 0.02) in the core, since the different environments where Bacillus activity takes place vary dramatically among species. Therefore, the mechanisms for sensing and responding to stimuli within each niche are not expected to be conserved but to be a part of the accessory genes. In contrast, the composition of COGs in the pan-genome shows similarity to that of the *B. cereus*-*anthracis*-*thuringiensis *group. This simply reflects the large redundancy of this group within the overall number of gene families (19,043 families) in the pan-genome.

*B. cereus, B. thuringiensis, B. anthracis, and B. weihenstephanensis *are described as members of a single species or taxon, the *B. cereus *group [43,44], and have the largest number of sequenced members as well as the largest genomes among known *Bacillus *(5,716 ± 354 coding genes). Therefore, it is remarkable that category G (carbohydrate transport and metabolism; *B. cereus *= 0.05/average = 0.07) is underrepresented compared to all other groups, while all other groups have similar amounts of genes from this category. This finding reflects a specialization in the metabolism of carbohydrates when compared to other groups. It is well documented that *B. cereus *has considerably less genes for the degradation of carbohydrates compared to *B. subtilis *[9], and this observation contradicts the hypothesis that the ancestor of *B. cereus *was a soil bacterium. The *B. cereus *group lacks the metabolic potential for the uptake and assimilation of plant-derived carbohydrates that exists in soil bacteria, such as *B. subtilis*, limiting the number of polysaccharides that are degraded by this group to glycogen, starch, chitin and chitosan [9]. The pathogenic *Bacillius *are included in the *B. cereus *group, and similar to the pan-genome, there is a clear predominance of genes in COG category V (involved in defense mechanisms; *B. cereus *= 0.03/average = 0.02), compared to a lower basal average for all of the *Bacillus *groups and the core genome. As expected, the largest repertoire of antibiotic resistance genes is present in the *B. cereus *group. This group is actively suffering selective pressure for these traits, a feature that is not observed in any of the other groups [33,45,46]. Restriction endonucleases, ABC-type transporters for the detoxification of cells, cation/multidrug efflux pumps, and enzymes involved in antibiotic resistance are all found within the COG V category. However, most pathogenic traits of these strains are encoded in plasmids and mobile elements. Examples of this are the Cry toxins of *B. thuringiensis*, the anthrax toxin and capsule genes of *B. anthracis *located on the pX01 and pX02 plasmids, and the emetic toxin of *B. cereus *located on the pX01-like plasmid (this feature has been used for phenotypic differentiation of the closely related strains). However, it has recently been shown that there can be multiple plasmid transfers among the *B. cereus *group strains [44], thereby compromising the main genetic and phenotypic differences within the cereus-*thuringiensis*-*anthracis *group.

Two interesting features appear when comparing the *B. coahuilensis*-m313-NRRLB14911 group to the other groups. First, this group contains the largest proportion of genes in category T (signal transduction; *B. coahuilensis*' group = 0.05/average = 0.04). These genes are thought to have been acquired through HGT, most likely from a *Cyanobateria*, in a similar manner as the sensory rhodopsin from *B. coahuilensis *[16]. The three members of this group were isolated from shallow waters exposed to high radiation and oligotrophic conditions. Two of them were isolated from a desiccation lagoon and the *Bacillus *sp. NRRLB14911 strain was isolated from the Gulf of Mexico at a depth of 10 m. These environments may require the strains to be responsive to sudden changes in conditions [15,16,26], and thus environmental sensing through signal transduction genes may be of greater importance than for other groups of *Bacillus*. This is a particularly noticeable feature of *B. coahuilensis*, a strain with a genome that seems to have undergone extensive size reduction [16]. The second interesting feature is that with exception to the *O. iheyensis *group, the *B. coahuilensis*-m313-NRRLB14911 group has fewer genes from category K (transcription factors; *B. coahuilensis *group = 0.08/average = 0.09) than all other groups. This group shares phenotypic traits, such as pigmentation, and are subject to a similar osmotic pressure due to salinity. This group also shows an underrepresentation of genes in category C (energy production and conversion; *B. coahuilensis *group = 0.05/average = 0.06), P (transport and metabolism of inorganic ions; *B. coahuilensis *group = 0.05/average = 0.06), and E (amino acid metabolism genes; *B. coahuilensis *group = 0.09/average = 0.1). All of these categories are involved in several of the early stages of amino acid synthesis. Several auxotrophies as well as specialization within these strains have been shown, particularly for *B. coahuilensis *[16]. This group exhibits the largest genome size variation with a range between 3.3 and 5 Mb. Despite the differences in the number of coding genes, the largest genome (that of NRRLB14911) does not bias the result of the COG T nor does it show an increase in transcription related genes. Finally, a larger than average gene content in the COG J category (translation, ribosome structure; *B. coahuilensis *group = 0.05/average = 0.06) is noticed in the group, which is congruent with the fact that they have an increase in the number of genes from COG L when compared with the *B. subtilis *group (replication, recombination and repair; *B. coahuilensis *group = 0.06/*B. subtilis *group = 0.06). We can hypothesize that the latter genes are needed to repair DNA that is damaged by the high radiation exposure. An over-representation of transposons and IS elements may be responsible for acquiring new genes via HGT or pseudogenization and the reduction of the genomes [47].

The *B. subtilis*-*licheniformis*-*pumilus *group has a slightly higher than average number of genes related to carbohydrate transport and metabolism (COG G; *B. subtilis *group = 0.08/average = 0.07) as expected for a group isolated from the soil and in close contact with plants and their products [2,9]. This group has a reduced number of genes involved in replication, recombination and repair (COG L) that correlates with the scarce repetitive elements such as IS, transposons, and transposases present in *B. subtilis*, *B. pumilus*, and other sister species [10]. Therefore, it seems that chromosome remodeling and genome reduction [48-50] is not a prevalent feature of *B. subtilis*. Interestingly, *B. pumilus *has a reduced number of genes involved in DNA repair and oxidative stress as well as small acid soluble proteins (SASP) that mitigate DNA damage and are involved in the desiccation and UV resistance of spores as compared to *B. subtilis *and close relatives [36,51]. In contrast, transcription-related genes (COG K; *B. subtilis *group = 0.1/average = 0.09) are slightly over-represented in the subtilis group. This suggests that the genetic response within this group is finely tuned as previously observed when the large gene families of *B. subtilis *and *B. coahuilensis *were compared [16].

*B. clausii-halodurans *form the group of alkalophiles and halotolerant strains. Interestingly, this group has fewer genes involved in the cell wall/membrane/envelope category (COG M; *B. clausii *group = 0.05/average = 0.06) than the average. This could be explained by the loss of 13 genes involved in the synthesis of teichoic acid and the loss of 6 genes involved in teichuronic acid biosynthesis. In addition, there are several known differences in the cell wall composition of *B. clausii-halodurans *compared to *B. subtilis*, such as the presence of the major cell wall component teichuronopeptide [14]. The number of genes in *B. clausii-halodurans *is similar to other *Bacillus *for the following categories: coenzyme transport and metabolism (COG H; *B. clausii *group = 0.03/average = 0.03), nucleotide transport and metabolism (COG F; *B. clausii *group = 0.03/average = 0.03), and protein turnover and chaperones (COG O; *B. clausii *group = 0.03/average = 0.03). The high number of genes from COG category G (carbohydrate metabolism; *B. clausii *group = 0.09/average = 0.07) stand out in this group. There are two possible explanations for this observation. First, *Bacillus *soil strains are expected to have a vast repertoire of genes for sugar assimilation, as is the case of *B. subtilis *[9], and *B. clausii *was isolated from soil [52]. Second, sugars, such as trehalose, function as osmoprotectants and therefore play an important role in halophilic bacteria [53]. The *B. clausii *genome has also an unusually high number of ABC transporter permeases (N = 36) that increase the number of genes within the COG G category. A noticeably high number of replication, recombination, and repair genes (COG L; *B. clausii *group = 0.07/average = 0.06) are present within this group. This may be due to the high number of repetitive elements in *B. halodurans*, which posseses 112 genes similar to transposases or recombinases [14] and numerous IS sequences [10]. Therefore, these repetitive elements may be considered as factors important for environment specialization [54-56].

*O. iheyensis *and *G. kaustophilus *were isolated from deep sea environments (1,050 and 3,000 m depth, respectively) but have different niche specializations. *O. iheyensis*, isolated from sediment and adapted to extreme salinity, is a facultative alkaliphilic. In contrast, *G. kaustophilus*, though also isolated from sediment, is associated with a marine trench and has an optimal growth temperature of 60°C. An important shared functional feature of this group is the resistance to osmotic pressure of up to 30 MPa, a unique characteristic when compared to normal atmospheric pressures of 0.1 MPa [10,14]. These two genomes are small in size (~3.6 Mb; see Table 1) and have less genes for several COG including nucleotide metabolism (COG F; *O. iheyensis *group = 0.03/average = 0.03). The essential genes of *B. subtilis*, such as *ymaA *and *ydiO*, are absent in *G. kaustophilus*[10]. There are only 12 genes in category Q for each of these genomes (secondary metabolism; *O. iheyensis *group = 0.01/average = 0.02), which is low when compared to an average of 14-21 genes per genome seen in category Q from other groups such as *B. cereus*. The number of genes categorized for transcription (COG K; *O. iheyensis *group = 0.08/average = 0.09) is also lower within this group and resembles the history of the *B. coahuilensis *group. The small genomes of this group and *B. coahuilensis *seem to be a result of genome reduction and adaptation to specific niches, such as oligotrophic environment, high salinity, high osmotic pressure, and thermal environments [47,54,56].

### Conservation of genes for competence and sporulation among members of *Bacillus*

Two post-exponential key processes have been subjected to extensive study for the genus *Bacillus*, and in particular the model system *B. subtilis*: genetic competence and sporulation. Genetic competence, defined as a state that permits the uptake of exogenous DNA, is widespread among both gram-positive and gram-negative bacteria. It is a genetically programmed state during which a small percentage of cells in a population can uptake DNA from the environment and integrate it into their chromosome [57]. Particularly, in the case of *B. subtilis *168, it is thought that the greater DNA uptake efficiency of the strain is a consequence of a laboratory selection process [2]. Most proteins that form the complex responsible for mediating the binding and uptake of DNA are part of the core (ComEC, ComFA, ComGA) or are highly conserved (ComEA). The interaction of several of these competence-specific proteins in the complex has been demonstrated, as well as interactions with the highly conserved proteins RecA, SsbB, and Smf [58]. The master regulator of the process, the ComK transcription factor, binds to competence promoters to activate transcription of many genes. A feature of competence development is the stabilization of ComK by protein ComS [59]. While the gene coding for the ComK transcription factor is conserved in most *Bacillus *(absent in *B. halodurans*, *B. clausii*, and *B. coahuilensis*), ComS seems to be a specialization of the *B. subtilis *group. ComK is itself synthesized in response to the signal-transduction network, but most genes coding for the regulatory proteins that constitute this network are not conserved. Given the conservation of the transformation machinery, it is of considerable interest to understand to what extent natural genetic competence can explain genetic variability by gene acquisition, at what frequency it occurs, and which signals trigger the competence state under specific environmental conditions.

A defining feature of the *Bacilli *is the formation of a highly resistant non-reproductive structure called the spore. Within the Firmicutes, the genus *Bacillus *and Clostridium produce endospores. The primary function of most spores is to ensure the survival of a bacterium through periods of environmental stress. The resistance of a spore could be considered as a crucial survival feature, and therefore sporulation genes would be expected to be part of the core genome. However, previous studies have found, both experimentally and *in silico*, that there is great intra-specific [2,60] and inter-specific variation in sporulation genes. The variability of sporulation genes has been only superficially reported in these studies and they have mainly included non-subtilis models such as *B. cereus *and *B. anthracis*.

The sporulation process has been subjected to extensive review [51,61-64]. This morphogenetic process is triggered by conditions of starvation that result in a distinct asymmetrically positioned septum that delimits the fore-spore and is surrounded by two membrane layers. Peptidoglycan is deposited in the space between the membranes to form the cortex and additional proteins are deposited around the cortex to form the so-called spore coat of the forming endospore. Some *Bacilli *also have an outer membrane composed of lipid and protein called an exosporium. The spore can quickly outgrow into a vegetative bacterium upon stimulation by an environmental cue.

**Figure 4:**
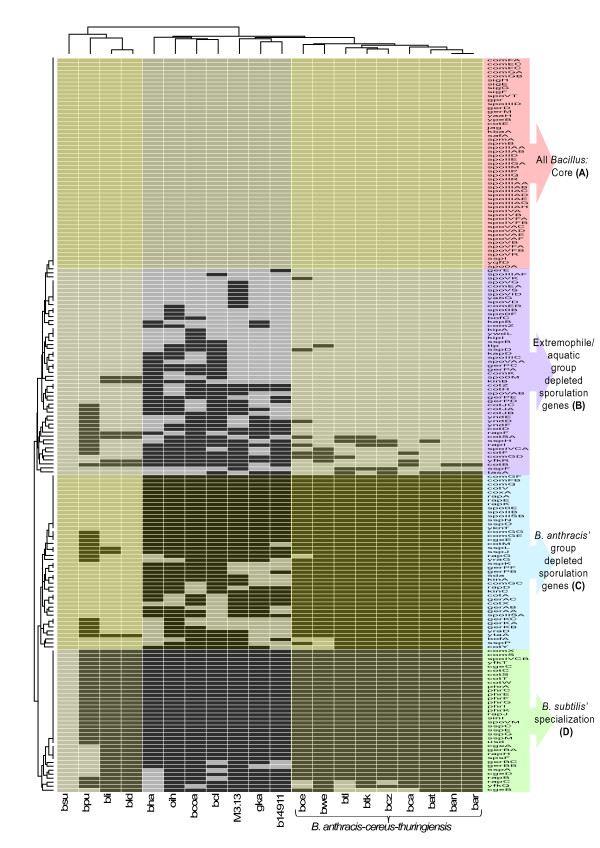
***Bacillus *selected sporulation and competence genes conservancy**. In this heat map shows the presence/absence of the set of 185 sporulation and competence related genes according to KEGG's BRITE hierarchies http://www.genome.jp/kegg/brite.html. Each column stands for a *Bacillus *strain and each row represents a gene and here are shown present (in gray) or absent genes (in black). Is possible to distinguish between 4 major clusters according to the conservancy level of the genes, (A) the first upper cluster stands for the sporulation core genes; (B) second is a group of sporulation genes diminished mostly in the extremophile and aquatic species of *Bacillus*; (C) cluster is defined as genes present in almost all the strains of *B. subtilis*' related group but depleted in the *B. cereus*' group; and (D) are *B. subtilis*' specialized sporulation genes. A comprensive list of each cluster of genes is available in Additional file 2: Table S3. Figure Abbreviations: *B. subtilis *(bsu), *B. pumilus*(bpu), *B. licheniformis *ATCC 14580 (bli), *B. licheniformis *DSM13 (bld), *B. halodurans *(bha), *O. iheyensis *(oih), *B. coahuilensis *(bcoa), *B. clausii *(bcl), *Bacillus *sp. m3-13 (M3.13), *G. kaustophilus *(gka), *Bacillus *sp.NRRLB-14911 (b14911), *B. cereus*ATCC 14579 (bce), *B. weihenstephanensis *(bwe), *B. thuringiensis *Al Hakam (btl), *B. thuringiensis*97-27 (btk), *B. cereus *ZK (bcz), *B. cereus *ATCC 10987 (bca), *B. anthracis *Sterne (bat), *B. anthracis *Ames (ban), *B. anthracis *Ames 0581 (bar).

Each of these stages has been documented and more than 200 regulatory and structural genes are known to be expressed in *B. subtilis *in a temporally regulated manner. Figure 4 depicts the conservation pattern of Kyoto Encyclopedia of Genes and Genomes' (KEGG) BRITE hierarchy [65] that comprises 185 sporulation related genes. These sporulation and germination genes are arguably the best studied transition regulators. Clustering of the conserved/absent sporulation and competence genes resulted in 4 clear groups that were arbitrarily denominated A through D. Group A, with 52 genes, contains all of the sporulation/competence core genes; group B, with 52 genes, shows great variability with an absence bias of genes in the extremophyle/aquatic *Bacillus*; Group C, with 43 genes, is conserved mainly among strains close to *B. subtilis*; and group D, with 35 genes, seems to represent the specialized genes of *B. subtilis*.

Within the sporulation core (Group A), we found genes required for the temporal and spatial regulation of sporulation gene expression that depends on four sporulation-specific sigma factors (SigE, F, G, and K), all of which are part of the core (the apparent lack of conservation of sigK is due to the fact that in *B. subtilis *it is encoded by two separate genes which are merged upon entrance into sporulation). As shown for *B. subtilis*, the initiation of sporulation is dependent on the phosphorylation of the two-component protein Spo0A, a transcription factor that controls a large number of genes. Spo0A is regulated through a phosphorylation cascade known as the phosphorelay. Sense input signals to Spo0A are given by histidine kinases, such as KinB, which are highly conserved (missing only in *O. iheyensis*). KbaA, the activator of KinB, is also present in the sporulation core. The number of sensor kinases that participate in this phosphorelay has been shown to differ between *B. subtilis *and *B. cereus *(5 and 9, respectively) [66]. The highly variable nature of the amino-terminal domains of the sporulation sensor kinases of the different *Bacillus *species has been suggested to represent differences in the signals used to initiate the developmental program. Recently, the *B. anthracis *kinase BA2291 was shown to be remarkably different from other sensor kinases by having a unique specificity for GTP [67]. Response regulator aspartate phosphatases as well as their cognate aspartate phosphatases are either not conserved or exhibit changes that make them difficult to recognize by sequence similarity. The only coat gene present in the core is cotE, which is involved in the outer layer of the spore coat [68]. Germination genes are also found within the core. GerD is involved in the early germination response to amino acids such as L-alanine and

L-asparagine [69], whereas GerM has been hypothesized to bind to peptidoglycan [70]. The spore maturation proteins SpmA and SpmB are also part of the sporulation core and are involved in spore dehydration. These proteins provide spore resistance to moist heat, as was shown in Clostridium perfringens [71,72]. Dipicolinic acid has been recognized as a core molecule that gives the spore radiation resistance [51]. The two units of dipicolinate synthase, spoVFA and spoVFB, are also present in the sporulation core. SspI appears to be the only universal SASP.

An interesting observation of group B is the seemingly generalized gene loss in the aquatic/halophile *Bacillus*. This finding is not seen in the *B. subtilis *and *B. anthracis*-cereus-*thuringiensis *groups. Diverse sporulation genes, germination genes, coat and small acid proteins are the predominant categories absent from this group. Variation of conservancy decreases with several SASPs, such as SspB, SspD, SspH, and SspF. This suggests that despite these proteins being very abundant in the spore, they are diverse and exhibit redundancy in places where they are bound to DNA (3-6% of the total spore protein) [51]. Therefore, the absence of some genes may be compensated by the presence of others.

The phosphatases RapF and RapI are involved in the phosphorelay and are poorly conserved across all *Bacillus*. The low conservation of phosphatase-related proteins is more prominent in groups C and D (RapA, B, C, D, E, G, and K;and PhrA, C, E, F, and G), suggesting that phosphorelay cascades may almost be strain-specific. A similar situation is observed for several coat proteins that first appear in group B (CotB, D, F, H, JA, JB, JC, SA, and Z) and for germination proteins (GerE, PA, PC, PD, and PE).

The cereus and subtilis group are similar in the A and B gene categories, but lack almost all genes described in categories C and D. Our results are consistent with previous studies [6-9,66] on comparative genomics within the *B. cereus *group that show that the main differences among the groups reside in HGT mobilized elements [31,73] and not in the core genome.

Our findings suggest that the sporulation sensor kinases, coat proteins, and SASPs of the various *Bacillus *species have evolved to be responsive to signals specific for particular environments. Even two very close strains, such as *B. licheniformis *DSM13 and ATCC 14580, differ in the preservation of some genes, including *sspJ *and *sspL*.

## Discussion

The increasing number of sequenced microbial genomes provides an ideal opportunity to re-evaluate approaches in understanding phylogenetic and functional differences among bacteria. Much of the understanding of microbial biodiversity has been studied by comparison of rRNA sequences. However, this approach has clear limitations, such as arbitrary cut-off values for sequence identity and the inability to resolve relationships between closely related groups [74]. For very close relatives, MLST and similar approaches can be used successfully to describe intra-diversity and resolve discrete clusters [75]. Both rRNA and MLST approaches use genes from the core genome [17], and in our analysis, we expanded the gene set for use in phylogenetic reconstruction in order to greatly increase our ability to resolve clusters. Testing inter-species phylogenetic cohesion of a group, such as the *Bacillus*, and taking advantage of 814 concatenated core genes, allowed us to obtain a robust phylogenetic reconstruction of the inner clusters that failed when comparing the same species with rRNA or universally conserved genes. Data obtained using metrics of taxa distance, such as GSS, for whole genome pairwise comparisons that made use of the entire shared genetic information agreed with the cluster resolution. Of note, we described the aquatic *Bacillus *as a new group. We predict that this group will quickly gain importance given the numerous examples of aquatic representatives that have been identified through 16S rRNA gene sequencing in multiple environmental samplings.

Today, given the constant improvement in cost/benefit of massive sequencing technologies, it is possible to think in whole genome shotgun (WGS) approaches to try to answer global internal group diversity. Although it is not yet the cheapest/feasible option for the regular laboratory, we suggest that the rapidly growing microbial genome database can be used to regularly and automatically build core genomes at intra-species, genus, family, order, and other taxonomic levels. This approach will aid in defining the functions of the genes behind different taxonomic ranks and provide the whole research community with specific genetic markers to perform detailed ecological and evolutionary analysis. Similar to the RBH approach, researchers can benefit from WGS projects in progress to define core genomes, since the number of unfinished genomes (1,777) almost doubles the amount of complete genomes (892). In this study, we used data from the sequenced genome of *Bacillus *sp. m3-13 with 22-fold coverage, the previously sequenced genome of *B*. *coahuilensis *[16], and the WGS assembly of *Bacillus *sp. NRRL-B14911 [15]. A core genome is a dynamic entity, since the incorporation of new genomes into the database will reduce the total number of genes within core genomes. How does core data compare with experimental data, such as the essential genes of *B. subtilis *[76]?. In this study, we found that the core genome for the sequenced *Bacillus *includes 61 of the 79 essential genes of *B. subtilis*. Another approach, using synteny [20] rather than RBH, defined 761 genes of the core genome. That particular study used several definitions of a core-genome and only considered complete genomes in the analysis. Our approach defined 814 genes and therefore obtained 54 more genes than the synteny approach. The use of a synteny strategy to identify a core genome would clearly miss rearranged orthologs and would also be limited if it were applied to WGS assemblies. Thus, working with orthologs as RBH may be a better approach.

Pan-genomes at genus level are only beginning to be described, and the number of gene families in these is expected to grow as more genomes are included. This approach however is highly valuable to reveal niche specific genes, to guide future studies linking genes to ecology, and even for selecting new genomes to sequence that could balance and enrich our knowledge of the *Bacillus *genus. The genetic features that explain metabolic diversity and that are part of the pan-genome and the non-core genes (known also as dispensable or "accessory" genes) are missing from the usual rRNA and MLST analysis. Genes involved in niche adaptation are amongst the dispensable genes and the whole pan-genome. Several of these genes are mobilized by means of HGT, such as genomic islands or individual elements involved in pathogenicity, as is the case for the *B. cereus *group [77], responses to environmental stresses like phosphorous deprivation found for *B. coahuilensis *[16]. In addition to core genome phylogenetics and the determination of ecological and geographical features, we suggest taking into account gene functions, such as COG comparative distributions, in order to describe species clusters and compare the phylogenetic profile with the functional profile. These data will aid in the analysis of the concordance or incongruence between them.

The existence of model organisms, such as *B. subtilis *str. 168, has been crucial for inferring homologous gene functions through all bacteria using physiological, genetic, and molecular biology approaches. The power and unequivocal value of the model organism in tackling biological mysteries is clear, but recent work on the intra-specific diversity of this model organism [78] has led us to recognize the great genomic variation that exists. These findings suggest that strain-specific genes are at the base of broad adaptations of *B. subtilis *to the terrestrial and aquatic environments from where it has been isolated. The *B. subtilis *str. 168 genome was sequenced in early 1997, but concerns regarding the effects of domestication of the strain in the laboratory and the subsequent genomic changes, as well as reevaluation of the quality of the original sequence, led to the re-sequencing of str. 168 [79]. Interestingly, other strains within the subtilis species show differences in the conservancy of genes. This is true even within the so-called essential genes, sporulation and competence genes, which are a central part of *Bacillus *biology. Therefore, the variability of gene content in the sporulation and essential genes amongst other representatives of the genus comes as no surprise. Although spore formation is central to the definition of *Bacilli*, it is clear that variability in the sensing of stress conditions, spore resistance, and germination is the result of specific niche constrains.

Use of comparative genomics, ecological and evolutionary facts, and the lowering costs of genomic sequencing are together aiding in the understanding of microbial diversity. Our next steps must focus on analyzing the temporal and spatial patterns of genes present or absent using high throughput genetic expression in order to understand the roles of microbes in their environments. Science is moving into a paradigm shift in the study of bacteria from single individuals to populations with the boost from metagenomic approaches. The challenges of unveiling comprehensive relationships between the environment, genes, and evolution of the bacterial species remain ahead.

## Conclusions

We have determined and defined a set of 814 genes that make up the core genome of *Bacillus*. From the core genome, we have reconstructed a robust phylogeny of the group using GSS index data, which use the total number of pairwise shared genes to resolve phylogenetic relationships within the group. Both the core genome and GSS phylogenies describe a new group of aquatic *Bacilli *that have similar habitats. To understand the biology of each group of *Bacillus *as defined by the respective phylogenies, we have described functional roles of their genes as well as differences between the core and pan-genomes. Our results show that a total of 53 genes comprise the sporulation and competence core genome. In addition, we have highlighted the differences in gene set conservancy across all *Bacillus *species that have been previously defined for *B. subtilis*. Our work will be a valuable resource for understanding the evolutionary and functional relationships within the *Bacillus *genus. The core genome defined here may also be used as a list of genetic markers for future population genetics studies. The lack of conservancy in non-*subtilis *groups of genes for processes such as sporulation and competence underlines the natural variability of the genus and emphasizes the need for further exploration of these differences.

## Methods

### Reciprocal Best Hits (RBH)

We undertook an RBH approach as previously described [80,81]in order to identify all of the orthologous pairs among 20 complete genomes of the *Bacillus*. Predicted, translated gene models for each genome were used and required coverage of 70% of both genes with an e-value of 10^-5 ^at an effective database size of 10^7^.

### Core genome

All pairwise RBH shared genes were compared and the common dataset of shared genes amongst all strains was defined as the core genome. We use the COG classification schema [41] to classify gene functions. The acronyms utilized in this study for the *B. subtilis *genes are the most widely used and we therefore used them for the entire core genome.

### Pan-genome

Here, we define the pan-genome as the total set of genes within the 20 *Bacillus genomes*, including plasmid and extra-chromosomal elements (when available). A total set of 19,043 homologous families were comprised of a total of 155,747 genes, as identified by RBH, with a cut-off e-value of 10^-5^[80]. COG classification was conducted with each representative from the homologous families.

### Evolutionary Analysis

To compute similarity between genomes using RBH pairwise information, we took advantage of our RBH bit-score results using a Genomic Similarity Score (GSS) [37]that had a range from 0 to 1.A maximum score was obtained when two compared proteomes were identical and a GSS distance matrix was used to build a Neighbor-Joining tree. Alignments for 16S rRNA were done using MUSCLE [82]and the phylogenetic tree was created using PhyML [83]. The 20 universally conserved gene COG phylogeny was constructed as previously described[16,40]. Each translated gene of the core genome was aligned and concatenated using ClustalW-MPI [84]. The phylogenetic tree was constructed using PhyML [83] with the JTT substitution model as has been done before with translated and concatenated sequences [40], given the alignment length (308,782 aa). The gamma distributed rates and 1,000 bootstraps were estimated from the dataset.

### Statistical analyses

All statistical analyses were conducted on R (2.6.2) [85]. Heat maps were generated with the gplots library of R [86].

### Sporulation genes

The genes were defined by the Kyoto Encyclopedia of Genes and Genomes KEGG BRITE Hierarchies [65]. RBH analysis, as previously described, was conducted with each KO gene annotated for *B. subtilis *in the BRITE hierarchy. RBH results were then parsed into presence/absence to map the conservancy of sporulation genes across the *Bacillus*.

### *Bacillus *sp. m3-13 isolation

The strain was isolated from a desiccation lagoon in the Churince system located in Cuatro Cienegas in Coahuila, Mexico (26°50.830'N, 102°09.335'W) by Rene Cerritos as described for *B. coahuilensis *[26].

### Genome sequencing, assembly, and annotation

*Bacillus *sp. m3-13 was sequenced using the 454 FLX system (454 Life Sciences) with a 20-fold coverage. Assembly was done with Newbler, Celera Assembler [87], and Phrap [88] resulting in 50 contigs and a total of 4,137,575 bp assembled. Gene prediction was done using Glimmer v3.0 [89] and GeneMark.hmm [90]. Automated annotation was performed with BASys [91]checked and proofed manually.

The Whole Genome Shotgun (WGS) *Bacillus *sp. m3-13 project has been deposited at DDBJ/EMBL/GenBank under the project accession [ACPC00000000]. The version described in this paper (ACPC01000000) is the first version.

## Abbreviations

CGH: Comparative Genome Hybridization; COG: Cluster of Orthologous Groups; GSS: Genome Similarity Score; HGT: Horizontal Gene Transfer; KEGG: Kyoto Encyclopedia of Genes and Genomes; ML: Maximum likelihood; MLSA: Multi Locus Sequence Analysis; MLST: Multi Locus Sequence Typing; RBH: Reciprocal Blast Hits; SASP: Small Acid Soluble Protein; WGS: Whole Genome Shotgun.

## Competing interests

The authors declare that they have no competing interests.

## Authors' contributions

LDA, GO, VS, and GM-H conceived and designed the study, LDA, GO, VS and LEE analyzed data, and LHE and GM-H contributed with reagents/materials/analysis tools. LDA, GO, and GM-H wrote the paper. All authors read and approved the final manuscript.

## Supplementary Material

Additional file 1 Core genome concatenate alignment in FASTA format. Click here for file

Additional file 2 **Supplementary tables**. **Table S1**. Current *Bacillus *sp. genome projects. **Table S2**. GI numbers, gene acronyms, and annotation for the 814 core genome genes. **Table S3**. Sporulation core genes. Click here for file

## References

[B1] Ravel J Fraser CM Genomics at the genus scale Trends Microbiol 2005 13 3 95 97 10.1016/j.tim.2005.01.004 15737726

[B2] Earl AM Losick R Kolter R Ecology and genomics of *Bacillus subtilis* Trends Microbiol 2008 16 6 269 275 10.1016/j.tim.2008.03.004 18467096 PMC2819312

[B3] Merrill L Dunbar J Richardson J Kuske CR Composition of *Bacillus *Species in Aerosols from 11 U.S. Cities Journal of Forensic Sciences 2006 51 3 559 565 10.1111/j.1556-4029.2006.00132.x 16696702

[B4] Hugenholtz P Exploring prokaryotic diversity in the genomic era Genome Biol 2002 3 2 REVIEWS0003 10.1186/gb-2002-3-2-reviews0003 11864374 PMC139013

[B5] Touchon M Hoede C Tenaillon O Barbe V Baeriswyl S Bidet P Bingen E Bonacorsi S Bouchier C Bouvet O Organised genome dynamics in the *Escherichia coli *species results in highly diverse adaptive paths PLoS Genet 2009 5 1 e1000344 10.1371/journal.pgen.1000344 19165319 PMC2617782

[B6] Anderson I Sorokin A Kapatral V Reznik G Bhattacharya A Mikhailova N Burd H Joukov V Kaznadzey D Walunas T Comparative genome analysis of *Bacillus cereus *group genomes with *Bacillus subtilis* FEMS Microbiol Lett 2005 250 2 175 184 10.1016/j.femsle.2005.07.008 16099605

[B7] Priest FG Barker M Baillie LWJ Holmes EC Maiden MCJ Population Structure and Evolution of the *Bacillus cereus *Group J Bacteriol 2004 186 23 7959 7970 10.1128/JB.186.23.7959-7970.2004 15547268 PMC529064

[B8] Helgason E Tourasse NJ Meisal R Caugant DA Kolsto A-B Multilocus Sequence Typing Scheme for Bacteria of the *Bacillus cereus *Group Appl Environ Microbiol 2004 70 1 191 201 10.1128/AEM.70.1.191-201.2004 14711642 PMC321270

[B9] Ivanova N Sorokin A Anderson I Galleron N Candelon B Kapatral V Bhattacharyya A Reznik G Mikhailova N Lapidus A Genome sequence of *Bacillus cereus *and comparative analysis with *Bacillus anthracis* Nature 2003 423 6935 87 91 10.1038/nature01582 12721630

[B10] Takami H Takaki Y Chee GJ Nishi S Shimamura S Suzuki H Matsui S Uchiyama I Thermoadaptation trait revealed by the genome sequence of thermophilic *Geobacillus kaustophilus* Nucleic Acids Res 2004 32 21 6292 6303 10.1093/nar/gkh970 15576355 PMC535678

[B11] Takami H Takaki Y Uchiyama I Genome sequence of *Oceanobacillus iheyensis *isolated from the Iheya Ridge and its unexpected adaptive capabilities to extreme environments Nucleic Acids Res 2002 30 18 3927 3935 10.1093/nar/gkf526 12235376 PMC137110

[B12] Kunst F Ogasawara N Moszer I Albertini AM Alloni G Azevedo V Bertero MG Bessieres P Bolotin A Borchert S The complete genome sequence of the gram-positive bacterium *Bacillus subtilis* Nature 1997 390 6657 249 256 10.1038/36786 9384377

[B13] Rey MW Ramaiya P Nelson BA Brody-Karpin SD Zaretsky EJ Tang M de Leon Lopez A Xiang H Gusti V Clausen IG Complete genome sequence of the industrial bacterium *Bacillus licheniformis *and comparisons with closely related *Bacillus *species Genome Biol 2004 5 10 R77 10.1186/gb-2004-5-10-r77 15461803 PMC545597

[B14] Takami H Nakasone K Takaki Y Maeno G Sasaki R Masui N Fuji F Hirama C Nakamura Y Ogasawara N Complete genome sequence of the alkaliphilic bacterium *Bacillus halodurans *and genomic sequence comparison with *Bacillus subtilis* Nucleic Acids Res 2000 28 21 4317 4331 10.1093/nar/28.21.4317 11058132 PMC113120

[B15] Siefert JL Larios-Sanz M Nakamura LK Slepecky RA Paul JH Moore ER Fox GE Jurtshuk P Jr Phylogeny of marine *Bacillus *isolates from the Gulf of Mexico Curr Microbiol 2000 41 2 84 88 10.1007/s002840010098 10856371

[B16] Alcaraz LD Olmedo G Bonilla G Cerritos R Hernandez G Cruz A Ramirez E Putonti C Jimenez B Martinez E The genome of *Bacillus coahuilensis *reveals adaptations essential for survival in the relic of an ancient marine environment Proc Natl Acad Sci USA 2008 105 15 5803 5808 10.1073/pnas.0800981105 18408155 PMC2311347

[B17] Tettelin H Masignani V Cieslewicz MJ Donati C Medini D Ward NL Angiuoli SV Crabtree J Jones AL Durkin AS Genome analysis of multiple pathogenic isolates of *Streptococcus agalactiae*: implications for the microbial "pan-genome" Proc Natl Acad Sci USA 2005 102 39 13950 13955 10.1073/pnas.0506758102 16172379 PMC1216834

[B18] Tettelin H Riley D Cattuto C Medini D Comparative genomics: the bacterial pan-genome Curr Opin Microbiol 2008 11 5 472 477 10.1016/j.mib.2008.09.006 19086349

[B19] Medini D Donati C Tettelin H Masignani V Rappuoli R The microbial pan-genome Curr Opin Genet Dev 2005 15 6 589 594 10.1016/j.gde.2005.09.006 16185861

[B20] Uchiyama I Multiple genome alignment for identifying the core structure among moderately related microbial genomes BMC Genomics 2008 9 515 10.1186/1471-2164-9-515 18976470 PMC2615449

[B21] Cortez D Forterre P Gribaldo S A hidden reservoir of integrative elements is the major source of recently acquired foreign genes and ORFans in archaeal and bacterial genomes Genome Biology 2009 10 6 R65 10.1186/gb-2009-10-6-r65 19531232 PMC2718499

[B22] Harris JK Kelley ST Spiegelman GB Pace NR The genetic core of the universal ancestor Genome Res 2003 13 3 407 412 10.1101/gr.652803 12618371 PMC430263

[B23] Dufresne A Ostrowski M Scanlan DJ Garczarek L Mazard S Palenik BP Paulsen IT de Marsac NT Wincker P Dossat C Unraveling the genomic mosaic of a ubiquitous genus of marine cyanobacteria Genome Biol 2008 9 5 R90 10.1186/gb-2008-9-5-r90 18507822 PMC2441476

[B24] Konstantinidis KT Tiedje JM Prokaryotic taxonomy and phylogeny in the genomic era: advancements and challenges ahead Curr Opin Microbiol 2007 10 5 504 509 10.1016/j.mib.2007.08.006 17923431

[B25] Gevers D Cohan FM Lawrence JG Spratt BG Coenye T Feil EJ Stackebrandt E Peer Y Van de Vandamme P Thompson FL Opinion: Re-evaluating prokaryotic species Nat Rev Microbiol 2005 3 9 733 739 10.1038/nrmicro1236 16138101

[B26] Cerritos R Vinuesa P Eguiarte LE Herrera-Estrella L Alcaraz-Peraza LD Arvizu-Gomez JL Olmedo G Ramirez E Siefert JL Souza V *Bacillus coahuilensis *sp. nov., a moderately halophilic species from a desiccation lagoon in the Cuatro Cienegas Valley in Coahuila, Mexico Int J Syst Evol Microbiol 2008 58 Pt 4 919 923 10.1099/ijs.0.64959-0 18398195

[B27] Breitbart M Hoare A Nitti A Siefert J Haynes M Dinsdale E Edwards R Souza V Rohwer F Hollander D Metagenomic and stable isotopic analyses of modern freshwater microbialites in Cuatro Cienegas, Mexico Environ Microbiol 2009 11 1 16 34 10.1111/j.1462-2920.2008.01725.x 18764874

[B28] Dyhrman ST Chappell PD Haley ST Moffett JW Orchard ED Waterbury JB Webb EA Phosphonate utilization by the globally important marine diazotroph Trichodesmium Nature 2006 439 7072 68 71 10.1038/nature04203 16397497

[B29] Van Mooy BAS Fredricks HF Pedler BE Dyhrman ST Karl DM Koblizek M Lomas MW Mincer TJ Moore LR Moutin T Phytoplankton in the ocean use non-phosphorus lipids in response to phosphorus scarcity Nature 2009 458 7234 69 72 10.1038/nature07659 19182781

[B30] Liolios K Mavromatis K Tavernarakis N Kyrpides NC The Genomes On Line Database (GOLD) in 2007: status of genomic and metagenomic projects and their associated metadata Nucleic Acids Res 2008 36 Database D475 479 17981842 10.1093/nar/gkm884 PMC2238992

[B31] Rasko DA Ravel J Okstad OA Helgason E Cer RZ Jiang L Shores KA Fouts DE Tourasse NJ Angiuoli SV The genome sequence of *Bacillus cereus *ATCC 10987 reveals metabolic adaptations and a large plasmid related to *Bacillus anthracis *pXO1 Nucl Acids Res 2004 32 3 977 988 10.1093/nar/gkh258 14960714 PMC373394

[B32] Read TD Peterson SN Tourasse N Baillie LW Paulsen IT Nelson KE Tettelin H Fouts DE Eisen JA Gill SR The genome sequence of *Bacillus anthracis *Ames and comparison to closely related bacteria Nature 2003 423 6935 81 86 10.1038/nature01586 12721629

[B33] Han CS Xie G Challacombe JF Altherr MR Bhotika SS Bruce D Campbell CS Campbell ML Chen J Chertkov O Pathogenomic Sequence Analysis of *Bacillus cereus *and *Bacillus *thuringiensis Isolates Closely Related to *Bacillus anthracis* J Bacteriol 2006 188 9 3382 3390 10.1128/JB.188.9.3382-3390.2006 16621833 PMC1447445

[B34] Ravel J Jiang L Stanley ST Wilson MR Decker RS Read TD Worsham P Keim PS Salzberg SL Fraser-Liggett CM The complete genome sequence of *Bacillus anthracis *Ames "Ancestor" J Bacteriol 2009 191 1 445 446 10.1128/JB.01347-08 18952800 PMC2612425

[B35] Challacombe JF Altherr MR Xie G Bhotika SS Brown N Bruce D Campbell CS Campbell ML Chen J Chertkov O The complete genome sequence of *Bacillus thuringiensis *Al Hakam J Bacteriol 2007 189 9 3680 3681 10.1128/JB.00241-07 17337577 PMC1855882

[B36] Gioia J Yerrapragada S Qin X Jiang H Igboeli OC Muzny D Dugan-Rocha S Ding Y Hawes A Liu W Paradoxical DNA Repair and Peroxide Resistance Gene Conservation in *Bacillus *pumilus SAFR-032 PLoS ONE 2007 2 9 e928 10.1371/journal.pone.0000928 17895969 PMC1976550

[B37] Moreno-Hagelsieb G Janga SC Operons and the effect of genome redundancy in deciphering functional relationships using phylogenetic profiles Proteins 2008 70 2 344 352 10.1002/prot.21564 17671982

[B38] Wolf YI Rogozin IB Grishin NV Tatusov RL Koonin EV Genome trees constructed using five different approaches suggest new major bacterial clades BMC Evol Biol 2001 1 8 10.1186/1471-2148-1-8 11734060 PMC60490

[B39] Wu D Hugenholtz P Mavromatis K Pukall R Dalin E Ivanova NN Kunin V Goodwin L Wu M Tindall BJ A phylogeny-driven genomic encyclopaedia of Bacteria and Archaea Nature 2009 462 7276 1056 1060 10.1038/nature08656 20033048 PMC3073058

[B40] Ciccarelli FD Doerks T von Mering C Creevey CJ Snel B Bork P Toward automatic reconstruction of a highly resolved tree of life Science 2006 311 5765 1283 1287 10.1126/science.1123061 16513982

[B41] Tatusov RL Fedorova ND Jackson JD Jacobs AR Kiryutin B Koonin EV Krylov DM Mazumder R Mekhedov SL Nikolskaya AN The COG database: an updated version includes eukaryotes BMC Bioinformatics 2003 4 41 10.1186/1471-2105-4-41 12969510 PMC222959

[B42] Price MN Dehal PS Arkin AP Orthologous Transcription Factors in Bacteria Have Different Functions and Regulate Different Genes PLoS Comput Biol 2007 3 9 e175 10.1371/journal.pcbi.0030175 PMC1971122 17845071

[B43] Helgason E Okstad OA Caugant DA Johansen HA Fouet A Mock M Hegna I Kolsto AB *Bacillus anthracis*, *Bacillus cereus*, and *Bacillus thuringiensis*--one species on the basis of genetic evidence Appl Environ Microbiol 2000 66 6 2627 2630 10.1128/AEM.66.6.2627-2630.2000 10831447 PMC110590

[B44] Hu X Auwera G Van der Timmery S Zhu L Mahillon J Distribution, Diversity, and Potential Mobility of Extrachromosomal Elements Related to the *Bacillus anthracis *pXO1 and pXO2 Virulence Plasmids Appl Environ Microbiol 2009 75 10 3016 3028 10.1128/AEM.02709-08 19304837 PMC2681636

[B45] Luna VA King DS Gulledge J Cannons AC Amuso PT Cattani J Susceptibility of *Bacillus anthracis*, *Bacillus cereus*, *Bacillus mycoides*, *Bacillus pseudomycoides *and *Bacillus thuringiensis *to 24 antimicrobials using Sensititre(R) automated microbroth dilution and Etest(R) agar gradient diffusion methods J Antimicrob Chemother 2007 60 3 555 567 10.1093/jac/dkm213 17586563

[B46] Schuch R Fischetti VA Detailed Genomic Analysis of the W{beta} and {gamma} Phages Infecting *Bacillus anthracis*: Implications for Evolution of Environmental Fitness and Antibiotic Resistance J Bacteriol 2006 188 8 3037 3051 10.1128/JB.188.8.3037-3051.2006 16585764 PMC1446989

[B47] Mira A Pushker R Rodriguez-Valera F The Neolithic revolution of bacterial genomes Trends Microbiol 2006 14 5 200 206 10.1016/j.tim.2006.03.001 16569502

[B48] Dufresne A Garczarek L Partensky F Accelerated evolution associated with genome reduction in a free-living prokaryote Genome Biol 2005 6 2 R14 10.1186/gb-2005-6-2-r14 15693943 PMC551534

[B49] Pushker R Mira A Rodriguez-Valera F Comparative genomics of gene-family size in closely related bacteria Genome Biol 2004 5 4 R27 10.1186/gb-2004-5-4-r27 15059260 PMC395786

[B50] Moran NA Mira A The process of genome shrinkage in the obligate symbiont Buchnera aphidicola Genome Biol 2001 2 12 RESEARCH0054 10.1186/gb-2001-2-12-research0054 11790257 PMC64839

[B51] Setlow P Spores of *Bacillus subtilis*: their resistance to and killing by radiation, heat and chemicals Journal of Applied Microbiology 2006 101 3 514 525 10.1111/j.1365-2672.2005.02736.x 16907802

[B52] Kageyama Y Takaki Y Shimamura S Nishi S Nogi Y Uchimura K Kobayashi T Hitomi J Ozaki K Kawai S Intragenomic diversity of the V1 regions of 16S rRNA genes in high-alkaline protease-producing *Bacillus *clausii spp Extremophiles 2007 11 4 597 603 10.1007/s00792-007-0074-1 17429572

[B53] Makihara F Tsuzuki M Sato K Masuda S Nagashima KV Abo M Okubo A Role of trehalose synthesis pathways in salt tolerance mechanism of *Rhodobacter sphaeroides *f. sp. denitrificans IL106 Arch Microbiol 2005 184 1 56 65 10.1007/s00203-005-0012-5 16052332

[B54] Mira A Pushker R The silencing of pseudogenes Mol Biol Evol 2005 22 11 2135 2138 10.1093/molbev/msi209 16014873

[B55] Mira A Klasson L Andersson SG Microbial genome evolution: sources of variability Curr Opin Microbiol 2002 5 5 506 512 10.1016/S1369-5274(02)00358-2 12354559

[B56] Mira A Ochman H Moran NA Deletional bias and the evolution of bacterial genomes Trends Genet 2001 17 10 589 596 10.1016/S0168-9525(01)02447-7 11585665

[B57] Chen I Dubnau D DNA uptake during bacterial transformation Nat Rev Micro 2004 2 3 241 249 10.1038/nrmicro844 15083159

[B58] Kramer N Hahn J Dubnau D Multiple interactions among the competence proteins of *Bacillus subtilis* Mol Microbiol 2007 65 2 454 464 10.1111/j.1365-2958.2007.05799.x 17630974

[B59] Maamar H Dubnau D Bistability in the *Bacillus subtilis *K-state (competence) system requires a positive feedback loop Mol Microbiol 2005 56 3 615 624 10.1111/j.1365-2958.2005.04592.x 15819619 PMC3831615

[B60] Earl AM Losick R Kolter R *Bacillus subtilis *genome diversity J Bacteriol 2007 189 3 1163 1170 10.1128/JB.01343-06 17114265 PMC1797320

[B61] Paredes CJ Alsaker KV Papoutsakis ET A comparative genomic view of clostridial sporulation and physiology Nat Rev Micro 2005 3 12 969 978 10.1038/nrmicro1288 16261177

[B62] Piggot PJ Hilbert DW Sporulation of *Bacillus subtilis* Curr Opin Microbiol 2004 7 6 579 586 10.1016/j.mib.2004.10.001 15556029

[B63] Hilbert DW Piggot PJ Compartmentalization of gene expression during *Bacillus subtilis *spore formation Microbiol Mol Biol Rev 2004 68 2 234 262 10.1128/MMBR.68.2.234-262.2004 15187183 PMC419919

[B64] Errington J Regulation of endospore formation in *Bacillus subtilis* Nat Rev Microbiol 2003 1 2 117 126 10.1038/nrmicro750 15035041

[B65] Kanehisa M Araki M Goto S Hattori M Hirakawa M Itoh M Katayama T Kawashima S Okuda S Tokimatsu T KEGG for linking genomes to life and the environment Nucl Acids Res 2008 36 suppl_1 D480 484 18077471 10.1093/nar/gkm882 PMC2238879

[B66] Perego M Kinase-phosphatase competition regulates *Bacillus subtilis *development Trends Microbiol 1998 6 9 366 370 10.1016/S0966-842X(98)01350-X 9778730

[B67] Scaramozzino F White A Perego M Hoch JA A unique GTP-dependent sporulation sensor histidine kinase in *Bacillus anthracis* J Bacteriol 2009 191 3 687 692 10.1128/JB.01184-08 18931112 PMC2632060

[B68] Bauer T Little S Stover AG Driks A Functional Regions of the *Bacillus subtilis *Spore Coat Morphogenetic Protein CotE J Bacteriol 1999 181 22 7043 7051 10559171 10.1128/jb.181.22.7043-7051.1999 PMC94180

[B69] Pelczar PL Igarashi T Setlow B Setlow P Role of GerD in Germination of *Bacillus subtilis *Spores J Bacteriol 2007 189 3 1090 1098 10.1128/JB.01606-06 17122337 PMC1797312

[B70] Daniel JR Michael YG Sequence analysis of GerM and SpoVS, uncharacterized bacterial sporulation proteins with widespread phylogenetic distribution Bioinformatics 2008 24 16 1793 1797 10.1093/bioinformatics/btn314 18562273 PMC2732212

[B71] Orsburn B Sucre K Popham DL Melville SB The SpmA/B and DacF proteins of *Clostridium perfringens *play important roles in spore heat resistance FEMS Microbiology Letters 2009 291 2 188 194 10.1111/j.1574-6968.2008.01454.x 19189487

[B72] Paredes-Sabja D Sarker N Setlow B Setlow P Sarker MR Roles of DacB and Spm Proteins in *Clostridium perfringens *Spore Resistance to Moist Heat, Chemicals, and UV Radiation Appl Environ Microbiol 2008 74 12 3730 3738 10.1128/AEM.00169-08 18441110 PMC2446547

[B73] Ehling-Schulz M Svensson B Guinebretiere M-H Lindback T Andersson M Schulz A Fricker M Christiansson A Granum PE Martlbauer E Emetic toxin formation of *Bacillus cereus *is restricted to a single evolutionary lineage of closely related strains Microbiology 2005 151 1 183 197 10.1099/mic.0.27607-0 15632437

[B74] Fraser C Alm EJ Polz MF Spratt BG Hanage WP The bacterial species challenge: making sense of genetic and ecological diversity Science 2009 323 5915 741 746 10.1126/science.1159388 19197054

[B75] Koeppel A Perry EB Sikorski J Krizanc D Warner A Ward DM Rooney AP Brambilla E Connor N Ratcliff RM Identifying the fundamental units of bacterial diversity: A paradigm shift to incorporate ecology into bacterial systematics Proceedings of the National Academy of Sciences 2008 105 7 2504 2509 10.1073/pnas.0712205105 PMC2268166 18272490

[B76] Kobayashi K Ehrlich SD Albertini A Amati G Andersen KK Arnaud M Asai K Ashikaga S Aymerich S Bessieres P Essential *Bacillus subtilis *genes Proc Natl Acad Sci USA 2003 100 8 4678 4683 10.1073/pnas.0730515100 12682299 PMC153615

[B77] Han CS Xie G Challacombe JF Altherr MR Bhotika SS Brown N Bruce D Campbell CS Campbell ML Chen J Pathogenomic sequence analysis of *Bacillus cereus *and *Bacillus *thuringiensis isolates closely related to *Bacillus anthracis* J Bacteriol 2006 188 9 3382 3390 10.1128/JB.188.9.3382-3390.2006 16621833 PMC1447445

[B78] Earl AM Losick R Kolter R Ecology and genomics of *Bacillus subtilis* Trends in Microbiology 2008 16 6 269 275 10.1016/j.tim.2008.03.004 18467096 PMC2819312

[B79] Barbe V Cruveiller S Kunst F Lenoble P Meurice G Sekowska A Vallenet D Wang T Moszer I Medigue C From a consortium sequence to a unified sequence: the *Bacillus subtilis *168 reference genome a decade later Microbiology 2009 155 Pt 6 1758 1775 10.1099/mic.0.027839-0 19383706 PMC2885750

[B80] Moreno-Hagelsieb G Latimer K Choosing BLAST options for better detection of orthologs as reciprocal best hits Bioinformatics 2008 24 3 319 324 10.1093/bioinformatics/btm585 18042555

[B81] Castillo-Ramirez S Gonzalez V Factors affecting the concordance between orthologous gene trees and species tree in bacteria BMC Evolutionary Biology 2008 8 1 300 10.1186/1471-2148-8-300 18973688 PMC2614993

[B82] Edgar RC MUSCLE: multiple sequence alignment with high accuracy and high throughput Nucleic Acids Res 2004 32 5 1792 1797 10.1093/nar/gkh340 15034147 PMC390337

[B83] Guindon S Gascuel O A simple, fast, and accurate algorithm to estimate large phylogenies by maximum likelihood Syst Biol 2003 52 5 696 704 10.1080/10635150390235520 14530136

[B84] Li KB ClustalW-MPI: ClustalW analysis using distributed and parallel computing Bioinformatics 2003 19 12 1585 1586 10.1093/bioinformatics/btg192 12912844

[B85] Team RDC Development Core Team, R: A language and environment for statistical computing 2008 R Foundation for Statistical Computing, Vienna, Austria

[B86] Gregory R Warnes BBaTL gplots: Various R programming tools for plotting data. R package version 2.6.0 2008

[B87] Miller JR Delcher AL Koren S Venter E Walenz BP Brownley A Johnson J Li K Mobarry C Sutton G Aggressive assembly of pyrosequencing reads with mates Bioinformatics 2008 24 24 2818 2824 10.1093/bioinformatics/btn548 18952627 PMC2639302

[B88] Ewing B Green P Base-calling of automated sequencer traces using phred. II. Error probabilities Genome Res 1998 8 3 186 194 9521922

[B89] Delcher AL Bratke KA Powers EC Salzberg SL Identifying bacterial genes and endosymbiont DNA with Glimmer Bioinformatics 2007 btm009 10.1093/bioinformatics/btm009 PMC2387122 17237039

[B90] Lukashin AV Borodovsky M GeneMark.hmm: new solutions for gene finding Nucl Acids Res 1998 26 4 1107 1115 10.1093/nar/26.4.1107 9461475 PMC147337

[B91] Van Domselaar GH Stothard P Shrivastava S Cruz JA Guo A Dong X Lu P Szafron D Greiner R Wishart DS BASys: a web server for automated bacterial genome annotation Nucleic Acids Res 2005 33 Web Server W455 459 10.1093/nar/gki593 15980511 PMC1160269

